# Cross-reactivity between apical membrane antgen 1 and rhoptry neck protein 2 in *P*. *vivax* and *P*. *falciparum*: A structural and binding study

**DOI:** 10.1371/journal.pone.0183198

**Published:** 2017-08-17

**Authors:** Brigitte Vulliez-Le Normand, Frederick A. Saul, Sylviane Hoos, Bart W. Faber, Graham A. Bentley

**Affiliations:** 1 Institut Pasteur, Unité de Microbiologie Structurale, Département de Biologie Structurale et Chimie, Centre National de la Recherche Scientifique, UMR 3528, Université Paris Diderot, Sorbonne Paris Cité, Microbiologie Structurale, Paris, France; 2 Institut Pasteur, Plate-forme de Cristallographie, Département de Biologie Structurale et Chimie, Centre National de la Recherche Scientifique UMR 3528, Paris, France; 3 Institut Pasteur, Plate-Forme de Biophysique Moléculaire, Département de Biologie Structurale et Chimie, Centre National de la Recherche Scientifique UMR 3528, Paris, France; 4 Department of Parasitology, Biomedical Primate Research Centre, Rijswijk, The Netherlands; 5 Institut Pasteur, Unité d’Immunologie Structurale, Département de Biologie Structurale et Chimie, Centre National de la Recherche Scientifique, URA 2185, Paris, France; Ehime Daigaku, JAPAN

## Abstract

Malaria, a disease endemic in many tropical and subtropical regions, is caused by infection of the erythrocyte by the apicomplexan parasite *Plasmodium*. Host-cell invasion is a complex process but two *Plasmodium* proteins, Apical Membrane Antigen 1 (AMA1) and the Rhoptry Neck protein complex (RON), play a key role. AMA1, present on the surface of the parasite, binds tightly to the RON2 component of the RON protein complex, which is inserted into the erythrocyte membrane during invasion. Blocking the AMA1-RON2 interaction with antibodies or peptides inhibits invasion, underlining its importance in the *Plasmodium* life cycle and as a target for therapeutic strategies. We describe the crystal structure of the complex formed between AMA1 from *P*. *vivax* (PvAMA1) and a peptide derived from the externally exposed region of *P*. *vivax* RON2 (PvRON2sp1), and of the heterocomplex formed between *P*. *falciparum* AMA1 (PfAMA1) and PvRON2sp1. Binding studies show that the affinity of PvRON2sp1 for PvAMA1 is weaker than that previously reported for the PfRON2sp1-PfAMA1 association. Moreover, while PvRON2sp1 shows strong cross-reactivity with PfAMA1, PfRON2sp1 displays no detectable interaction with PvAMA1. The structures show that the equivalent residues PvRON2-Thr2055 and PfRON2-Arg2041 largely account for this pattern of reactivity.

## Introduction

Invasion of the erythrocyte by the malaria parasite, *Plasmodium*, follows a sequence of distinct steps [1]. During the first step, the randomly oriented merozoite (the blood-stage form of *Plasmodium*) makes loose contacts with the erythrocyte. This initial association is reversible as the parasite may subsequently disengage from the host cell. The merozoite then reorients to bring the apical pole into contact with the erythrocyte membrane, creating a strong irreversible attachment. Finally, the moving junction (MJ), a ring-like structure formed between the erythrocyte and merozoite membranes, moves over the entire merozoite surface from the apex to the posterior pole, completely invaginating the parasite within the host cell [2]. Upon completion of invasion, the parasite, now contained within the parasitophorous vacuole inside the erythrocyte, begins the intra-erythrocytic phase leading to asexual replication.

A number of proteins, either exposed on the merozoite surface or stored in the apical organelles, are important for this intricate invasion process [3]. Two of these, Apical Membrane Antigen 1 (AMA1) and the Rhoptry Neck (RON) complex comprising RON2, RON4 and RON5, play a crucial role at the MJ. [4,5]. AMA1 is transported from the microneme organelles to the merozoite surface prior to invasion [6], while the RON complex, initially stored in the rhoptries, inserts into the host cell membrane in a coordinated secretion process at the start of invasion [7], as has been shown with the closely related apicomplexan parasite *Toxoplama gondii* [8,9]. In this invasion model, AMA1 and the RON complex then bind together linking the merozoite and erythrocyte membranes to form an essential structural component that maintains the MJ ring. Thus, *Plasmodium* (and probably most other apicomplexan parasites) provides its own host-cell receptor for AMA1.

Recent structural studies have shown that, in *P*. *falciparum* and *T*. *gondii*, AMA1 binds to the extracellular region of the RON2 component bounded by the second and third putative transmembrane regions [10,11]. The receptor-binding site on *P*. *falciparum* and *T*. *gondii* AMA1 (PfAMA1 and TgAMA1, respectively) comprises a region on Domain 1 of the ectoplasmic domain termed the hydrophobic groove and a region immediately adjacent that becomes exposed when the mobile Domain 2 (D2) loop (residues Ser347-His393) is displaced by the incoming ligand. Blocking the interaction between PfAMA1 and PfRON2 by antibodies [4,12] or peptides [4, 13, 14] inhibits invasion, confirming that the AMA1-RON2 interaction is essential in this stage of the *Plasmodium* life cycle. Crystal structure studies have shown that homologous binding sites also exist on apo AMA1 from *P*. *vivax* [15] and *P*. *knowlesi* [16], as well as from the apicomplexan parasites *Babesia divergens* (Bd) and *Neospora caninum* (Nc) [17]. Furthermore, binding studies show that BdAMA1 and NcAMA1 bind to the extracellular region of their respective RON2 homologues [17], suggesting that this mechanism is most likely common to the *Apicomplexa* phylum.

Here we describe structural and binding studies that extend our knowledge of the AMA1-RON2 interaction within the *Plasmodium* genus. We have determined the crystal structure of AMA1 from *P*. *vivax* (PvAMA1) in complex with a 39-residue peptide corresponding to the extracellular region of RON2 from *P*. *vivax* (PvRON2sp1) and, in addition, the structure of the cross-reaction complex formed between PvRON2sp1 and PfAMA1. Although the structure of PvRON2sp1 is very similar in the homo- and hetero-complexes, differences show how the ligand adjusts to structural variations in the binding sites of the two AMA1 homologues. In particular, PvRON2sp1 from the heterocomplex is structurally closer to PfRON2sp1 in the previously determined PfAMA1-PfRON2sp1 structure [10] than in the *P*. *vivax* homocomplex. Binding measurements by surface plasmon resonance (SPR) show that PvRON2sp1 binds to PvAMA1 with a K_D_ of 50nM whereas its affinity to PfAMA1 is 3- to 4-fold weaker. By contrast, PfRON2sp1 shows no detectable affinity to PvAMA1. Comparison of the crystal structures of AMA1-RON2 complexes provides a basis for understanding these different cross-reactive behaviours and may aid the development of novel molecules that block this crucial interaction in the invasion mechanism of *Plasmodium*.

## Methods and materials

### Recombinant protein production

PvAMA1 Sal-1 strain (residues 43–487; Domains 1, 2 and 3), followed by c-myc epitope and hexa-histidine tags) was produced in *Pichia pastoris* as previously described [18]. PfAMA1 strain FVO (residues 97–442; Domains 1 and 2), followed by c-myc epitope and hexa-histidine tags) used for crystallographic studies, and strains FVO and 3D7 (residues 25–545; Domains 1, 2 and 3), no tag) used for SPR experiments were expressed as described previously [19, 20].

### Peptide synthesis

PvRON2sp1 and PfRON2sp1, 39-residue peptides corresponding to Met2034-Leu2072 of PvRON2 and Met2020-Leu2058 of PfRON2, respectively, were synthesized with the disulphide bonds (Cys2051-Cys2063 for PvRON2sp1 and Cys2037-Cys2049 for PfRON2sp1). Synthesis was performed by Polypeptide (Strasbourg, France) with purity levels of 70% for PvRON2sp1 and 92% for PfRON2sp1. The peptides were solubilized in 5% DMSO for subsequent use.

### Binding studies by SPR

SPR measurements were made with a Biacore 2000 instrument (Biacore, AB). The Pv AMA1 and PfAMA1 proteins, diluted in 10 mM sodium acetate pH 4.5, were covalently immobilized (2000–2500 resonance units (RU)) by an amine-coupling procedure on CM5 sensor chips (GE Healthcare). The reference flow cell was prepared by the same procedure in absence of protein. Binding assays were performed at 25°C in PBS and 0.005% Tween 20 by injecting a series of PvRON2sp1 peptide concentrations at a constant flow rate of 5 μl/min. A heterologous peptide was used to verify the absence of non-specific binding. Peptide dissociation was measured by injecting the running buffer and the surface was regenerated by injecting glycine/HCl pH 2, followed by SDS 0.05%. Control flow cell sensorgrams were subtracted from the ligand flow cell sensorgrams and averaged buffer injections were subtracted from analyte sensorgrams. Estimated values of equilibrium response level (Req) were obtained by extrapolation from the experimental curves since the association phase did not reach a final equilibrium state. All calculations were made using the BIAevaluation 4.2 software (BIAcore AB). The saturation curves obtained by plotting Req versus the peptide concentration were fitted with a steady-state model to obtain the Rmax and the apparent equilibrium dissociation constants, K_D_. To normalize the response for the different ligands, these curves were reported as the percentage of bound sites (ratio Req/Rmax) versus the analyte concentration.

### Crystallization and data collection

Crystals of the complex PvAMA1-PvRON2sp1 were grown over 17% PEG 3350, 85 mM sodium citrate pH 5.6, 17% propanol-2, and the protein was incubated with PvRON2sp1 (1:5.8 ratio). Crystals of PfAMA1(FVO)-PvRON2sp1 were obtained over 8% PEG3350, 100 mM Tris pH 8,2, 100 mM sodium acetate and 20% propanol-2 with the protein incubated in a ratio 1:4 with the peptide. Crystals in cryoprotectant were flash cooled at 100 K and diffraction data were collected on beamline PROXIMA 1 at synchrotron SOLEIL (St Aubin, France).

### Data, processing, structure solution and refinement

Diffraction data were processed with the programs XDS [21] and SCALA [22] in the CCP4 suite of programs [23]. Crystallographic parameters and data collection statistics are given in Table 1. For each complex, initial phases were obtained by molecular replacement using PHASER [24]. The search models used were the unliganded PvAMA1 structure (PDB entry 1W8K) for the PvAMA1-PvRON2sp1 complex, and the PfAMA1(3D7)-PfRON2sp1 structure (PDB entry 3ZWZ) for the PfAMA1(FVO)-PvRON2sp1 complex. The structures were refined with the program BUSTER [25]. The PvRON2sp1 peptide was built into the electron density of each complex as refinement progressed.

**Table 1:** Crystallographic parameters and data collection statistics.

	PvAMA1-PvRON2sp1	PfAMA1(FVO)-PvRON2sp1
**Crystal parameters**		
Spacegroup	C2	P2_1_
a, b, c (Å)	164.46, 54.00, 62.13	71.48, 38.25, 72.36
β (°)	105.71	98.55
*V*_M_ (A^3^ /Da^-1^)	2.27	2.09
**Data Statistics**		
Resolution limits (Å)	42.50–2.15 (2.27–2.15)	46.92–1.90 (2.00–1.90)
R_merge_	0.087 (0.750)	0.083 (0.569)
R_pim_	0.052 (0.452)	0.056 (0.497)
Unique reflections	28677 (4162))	29,741 (3444))
Multiplicity	3.7 (3.7)	3.0 (1.8)
Completeness (%)	99.6 (99.8)	96.2 (78.3)
Wilson B-factor (Å^2^)	40.1	23.3
<I*/*σ(I)>	9.6 (1.9)	9.2 (1.4)
CC_1/2_	0.997 (0.698)	0.996 (0.822)

Values in parentheses are for the highest resolution shell.

A summary of refinement statistics is given in Table 2. All structural figures were generated with Pymol [26]. Coordinates and structure factors have been deposited in the Protein Data Bank with the following entry codes: PvAMA1-PvRON2sp1 complex 5NQG and PfAMA1(FVO)-PvRON2sp1 complex 5NQF.

**Table 2:** Refinement statistics.

	PvAMA1-PvRON2sp1	PfAMA1(FVO)-PvRON2sp1
Resolution (Å)	39.6–2.15 (2.23–2.15)	35.8–1.90 1.97–1.90)
R value, working set	0.174 (0.229)	0.169 (0.233)
R_free_	0.226 (0.249)	0.205 (0.295)
No of reflections	28038 (2361)	28038 (2361)
No. atoms protein/solvent	3324/234	2626/307
**rms deviation from ideal**		
Bond length (Å)	0.010	0.010
Bond angle (°)	1.09	1.04
**Ramachandran plot**		
Preferred regions (%)	95.8	96.5
Allowed regions (%)	3.2	2.9
Outliers (%)	1.0	0.7

Values in parentheses are for the highest resolution shell.

## Results and discussion

### Binding measurements of PvRON2sp1 to PvAMA1 and PfAMA1

**Fig 1:**
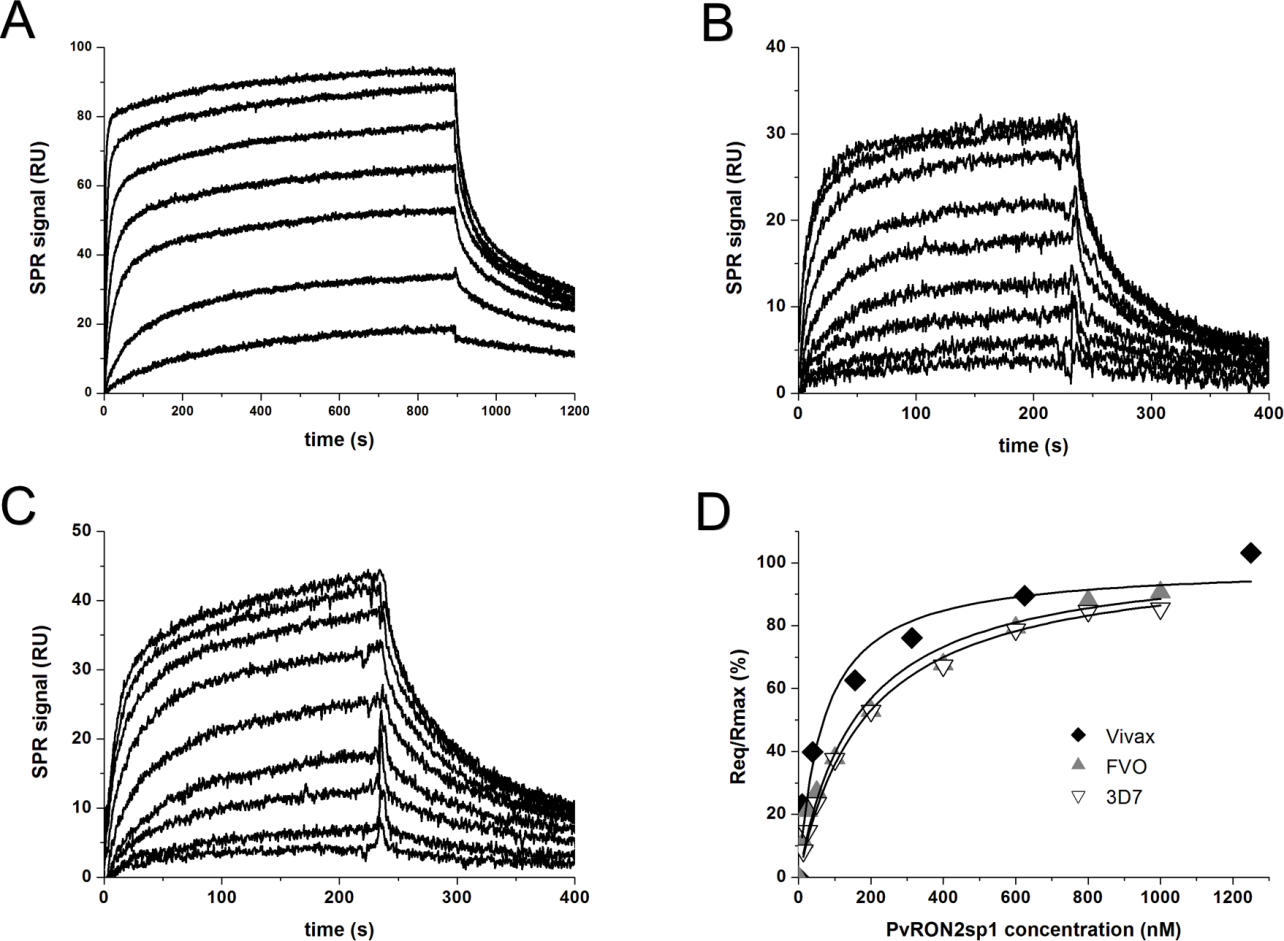
Binding measurements of PvRON2sp1 to PvAMA1 and PfAMA1 using surface plasmon resonance. Sensograms for the binding of PvRON2sp1 to (A) PvAMA1, (B) PfAMA1 (FVO strain), and (C) PfAMA1 (3D7 strain). (D) Steady state binding curves showing the fraction of bound sites as a function of PvRON2sp1 concentration. The following peptide concentrations were injected: 10, 39, 156, 313, 625, 1250, 2500 nM for PvAMA1, and 12.5, 25, 50, 100, 200, 400, 600, 800 and 1000 nM for 3D7 and FVO.

The binding constants, K_D_, of PvRON2sp1 to PvAMA1 (strain Sal-1) and to PfAMA1 (strains FVO and 3D7), were determined from the variation of the steady state SPR amplitude with peptide concentration. PvRON2sp1 binds to PvAMA1 with a K_D_ of 50 nM and to PfAMA1 with an affinity 3–4 times lower (depending on the *P*. *falciparum* strain) (Fig 1, Table 3). These interactions are weaker than those previously found for the binding of PfRON2sp1 to different strains of PfAMA1, where K_D_ ranged from about 10–20 nM [10]. By contrast, attempts to measure binding of PfRON2sp1 by PvAMA1 failed to show any detectable response (S1 Fig).

**Table 3:** Binding constants determined by SPR. K_D_ was determined from the steady-state binding amplitude as a function of the PvRON2sp1 peptide concentration. The values given are the mean from at least two independent experiments with the standard deviation shown in parenthesis.

Analyte/Ligand Immobilized	K_D (nM)_
PvRON2sp1/PvAMA1	50 (17)
PvRON2sp1/PfAMA1(FVO)	169 (29)
PvRON2sp1/PfAMA1(3D7)	199 (27)

### General description of the AMA1-RON2 structures

Crystals were obtained for complexes formed between PvAMA1 strain Sal-1 (comprising domains D1, D2 and D3), or PfAMA1 strain FVO (comprising domains D1 and D2), and the peptide PvRON2sp1 (39 residues: Met2034 to Leu2072; PvRON2 residue numbering from PlasmoDB entry PVX_117880). The PvRON2sp1 peptide is bounded by two putative transmembrane regions (residues 2016–2034 and 2089–2209, [27] and, by analogy with *T*. *gondii* [8], is considered to be exposed externally when the RON2 protein is embedded in the erythrocyte membrane.

#### (i) Homocomplex PvAMA1-PvRON2sp1

**Fig 2:**
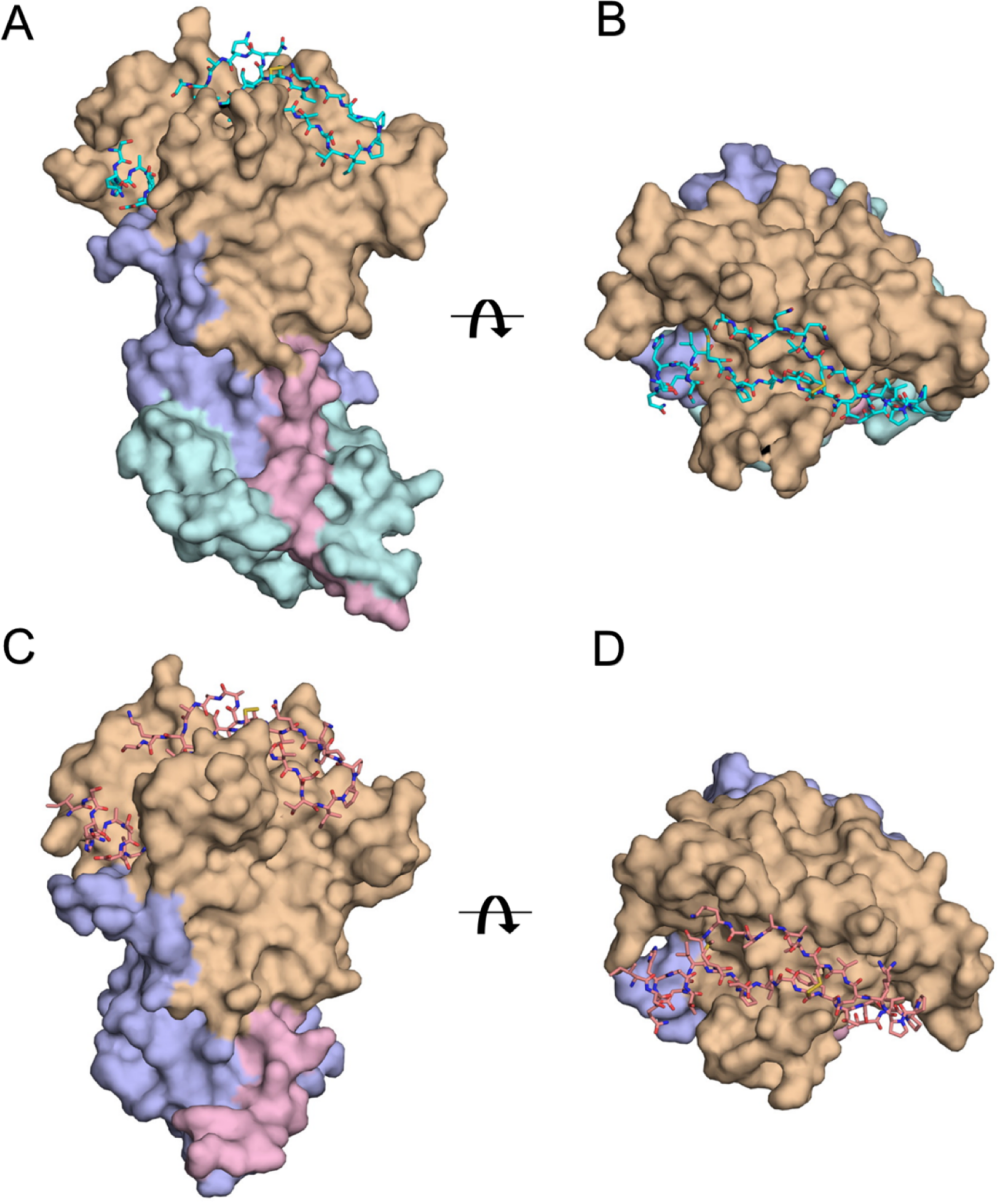
Comparison between the homo-complex PvAMA1-PvRON2sp1 and the hetero-complex PfAMA1-PvRON2sp1. Two orthogonal views of the complexes are shown: (A), (B) The PvAMA1-PvRON2sp1 complex is shown in surface representation with the N-terminal extension in pink (residues 43 to 62), Domain I in light brown (residues 63 to 248), Domain II in mauve (residues 249 to 385) and Domain III in cyan (residues 386 to 474); PvRON2sp1 is shown in stick representation. (C), (D) The PfAMA1-PvRON2sp1 complex is shown with the same relative orientations as in A and B, respectively. PfAMA1 is shown in surface representation with the N-terminal extension in pink (residues 102 to 123), Domain I in light brown (residues 124 to 303), Domain II in mauve (residues 304 to 440); PvRON2sp1 is shown in stick representation.

The refined structure of the complex includes residues Pro43 to Tyr474 of PvAMA1 with the following gaps in the polypeptide chain tracing: Gln297-Asn334 (the D2 loop, which is very mobile in many AMA1 structures of different *Plasmodium* species [15, 16, 28], and Gln402-Gly413. A total of 33 of the 39 residues of the bound PvRON2sp1 ligand could be followed in the electron density from Ser2037 to Lys2069 (Fig 2A and 2B).

The PvRON2sp1 peptide occupies a groove of PvAMA1 that is located mostly on domain D1 and contributes the majority of the contacts; however, loop regions surrounding the groove also contribute to the binding. The peptide is boomerang-shaped with one wing formed by a disulphide-bridged β-hairpin loop comprising the central segment of the peptide between residues Cys2051 to Cys2063, while the other wing is formed by the flanking N- and C-terminal segments. The solvent-inaccessible surface at the PvAMA1-PvRON2sp1 buried at interface is 2893 Å^2^.

The PvAMA1 component of the complex is very similar in structure to the apo protein (1W8K), with a r.m.s.d. of 1.11 Å over the 359 common Cα atoms but some important structural changes occur on binding PvRON2sp1. The loop 129–134 undergoes a significant conformational change upon binding PvRON2sp1, with Asp133 moving by more than 6 Å, and the loop 171–176, which is completely disordered in the apo PvAMA1, makes contacts with PvRON2sp1 and could be completely traced in the electron density of the complex. The loop 148–151 also contacts the peptide ligand but shows no important structural differences with respect to that of the free PvAMA1. Finally, residues 296 and 297 at the N-terminal base of the disordered D2 loop contact PvRON2sp1.

#### (ii) Heterocomplex PfAMA1-PvRON2sp1

The heterocomplex PfAMA1-PvRON2sp1 was crystallized using recombinant PfAMA1, comprising domains D1 and D2 only, from strain FVO. The PfAMA1(FVO) polypeptide chain was traced from Arg102 to Pro442 with gaps Gly259-Arg261, Lys265-Ser268, Tyr353-Asp388 (the D2 loop); 33 of the 39 residues of the PvRON2sp1 peptide were traced from Ser2037 to Lys2069. The PfAMA1(FVO) heterocomplex is shown in Fig 2C and 2D.

As with the homocomplex, the bound peptide in the PfAMA1(FVO)-PvRON2sp1 heterocomplex adopts a boomerang shape. A disulphide-bridged β-hairpin formed by the central segment between the two cysteine residues constitutes one wing, and the flanking N- and C-terminal segments form the other wing. The buried surface at the PfAMA1(FVO)-PvRON2sp1 interface of the heterocomplex is 3115 Å^2^, larger than that of the *P*. *vivax* homocomplex. By comparison, the buried surface in the *P*. *falciparum* homocomplex (PDB entry 3ZWZ) is larger (3224 Å^2^). This difference can be mainly attributed to the more important contribution by Arg2041 of PfRON2 in comparison to the equivalent residue Thr2055 of PvRON2.

The PfAMA1 component in the heterocomplex is similar in structure to apo PfAMA1(3D7) [28] (PDB entry 1Z40, 1.08 Å r.m.s.d. over 274 Cα positions) and even closer to that of the antigen in the PfAMA1(3D7)-PfRON2sp1 homocomplex (PDB entry 3ZWZ, 0.86 Å r.m.s.d. over 286 Cα positions). The structural differences between PfAMA1(FVO) of the heterocomplex and apo PfAMA1(3D7) are, as with the PvAMA1-PvRON2sp1 complex, largely confined to some of the loop segments that contact the ligand. The loop 172–176, which is disordered in the apo PfAMA1(3D7), could be completely traced in the heterocomplex, revealing a conformation closely matching that found in the PfAMA1 homocomplex. Loops 185–190 and 202–207 contact the PvRON2sp1 peptide but show no significant structural differences with respect to this region of apo PfAMA1(3D7) (or, indeed, the homologous segment of apo PvAMA1). The loop 226–233 is displaced by more than 5 Å upon forming the complex and is essentially identical in conformation to this segment in the PfAMA1-PfRON2sp1 homocomplex. The loop 264–274, which is disordered in apo PfAMA1, contacts the N-terminal region of PvRON2sp1 but could only be partially traced. This PfAMA1 segment was also completely traced in the PfAMA1-PfRON2sp1 homocomplex [10].

### Comparison of the RON2 ligands

**Fig 3:**
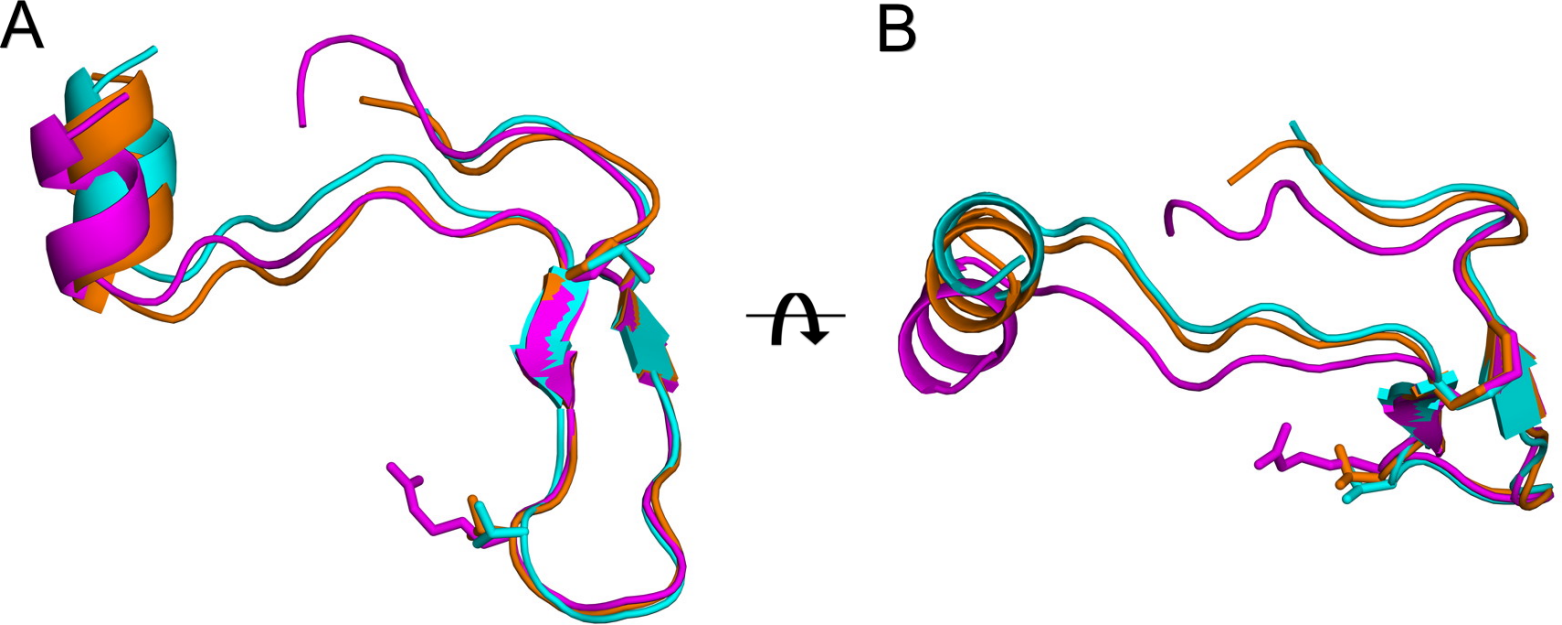
Comparison of the PvRON2sp1 peptides in the homo- and hetero-complexes, and PfRON2sp1 in the homo-complex PfAMA1-PfRON2sp1. PvRON2sp1 is shown in cyan for the homo-complex and in orange for the hetero-complex; PfRON2sp1 from the PfAMA1-PfRON2sp1 homo-complex (PDB entry 3ZWZ) is shown in magenta. (A) The three ligands, shown in ribbon representation, were superimposed using the main-chain coordinates of the disulphide-bridged β-hairpin (Cys2051-Cys2063 in PvRON2sp1, Cys2037-Cys2049 in PfRON2sp1). The side chains of Arg2041 of PfRON2sp1, which makes critical contacts with PfAMA1 in the *P*. *falciparum* homo-complex, and of the equivalent residue in PvRON2sp1, Thr2055, are also shown. The superimposed ligands are oriented such that the N-terminal helix of PvRON2sp1 in the homo-complex (cyan) is vertical. (B) View of the superimposed ligands rotated by 90° about the horizontal axis.

**Fig 4:**
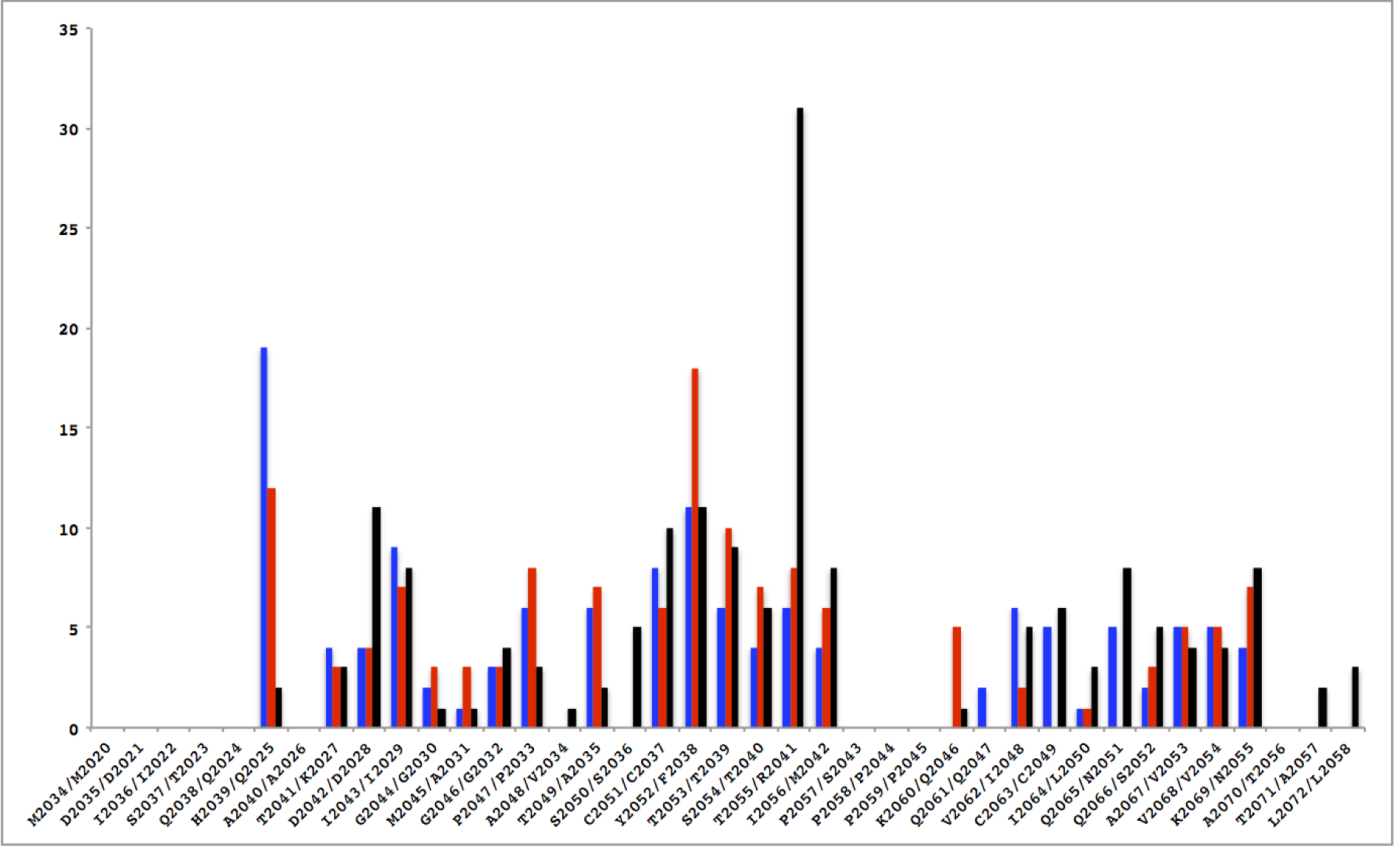
Comparison of contacts between AMA1 and the RON2 peptide in the homo- and hetero-complexes. The number of interatomic contacts < 3.8 Å (ordinate) is given as a function of the RON2 sequence (abscissa; PvRON2/PfRON2). In the histogram, the PvAMA1-PvRON2sp1 complex is shown in blue, the PfAMA1(FVO)-PvRON2sp1 complex is in red and the PfAMA1(3D7)-PfRON2sp1 complex [10] is in black.

The PvRON2sp1 peptide in complex with PvAMA1 or PfAMA1 reported in this study, and PfRON2sp1 in the previously determined homocomplex PfAMA1-PfRON2sp1 structure [10], are very similar in conformation (Fig 3, Table 4). In the homo- and hetero-complex with PvRON2sp1 as ligand, the N-terminal segment of the bound peptide begins with a short α-helix from residues Ser2037 to Ile2043, followed by an extended peptide stretch to a disulphide-linked β-hairpin formed between Cys2051 and Cys2063. The C-terminal region, following Cys2063, is extended but with no regular structural elements. This structure is very similar to that of PfRON2sp1 when bound to PfAMA1: the N-terminal segment begins with a short α-helix from the equivalent residues Gln2024 to Ile2029, followed by an extended peptide conformation to a disulphide-bridged β-hairpin formed between Cys2037 and Cys2049. The C-terminal segment after Cys2049 follows a similar conformation to that of PvRON2sp1. The disulphide-bridged β-hairpin, which forms one wing of the boomerang-shaped peptide, is the region that shows closest structural similarity between the RON2 peptides in the three complexes (Fig 3). Nonetheless, small variations in conformation of PvRON2sp1 between the homo- and hetero-complex suggest that the peptide adapts to structural differences between the binding sites of PvAMA1 and PfAMA1; the conformation of PvRON2sp1 in the heterocomplex is closer to that of PfRON2sp1 in the *P*. *falciparum* homocomplex (r.m.s.d. 0.70 Å in main-chain atoms) than to the *P*. *vivax* homocomplex (0.82 Å). The pattern of contacts between AMA1 and RON2 is similar in the three complexes with some of the polar contacts being conserved. Differences do occur, however, because of sequence variations between the two species, both for receptor and ligand (Fig 4, S1 Table).

**Table 4:** Comparison of PvRON2sp1 and PfRON2sp1 in complex with PvAMA1 or PfAMA1. The r.m.s.d in the main-chain atoms over the 33 common residues is given in a pairwise comparison.

	PvRON2 (PfAMA1-FVO heterocomplex)	PfRON2 (PfAMA1-3D7 homocomplex)
PvRON2 (PvAMA1 homocomplex)	0.82	0.96
PvRON2 (PfAMA1-FVO heterocomplex)	-	0.70

The N-terminal helices of the RON2 ligands are in very close coincidence when the AMA1 structures of the three complexes are superimposed. Interestingly, this coincidence extends to the N-terminal helix of TgRON2 (which comprises residues Asp1297 to Ile1305 and is thus longer than in the *Plasmodium* homologues) in complex with TgAMA1, the only other such AMA1-RON2 structure determined to date (S2 Fig). By contrast, the disulphide-linked β-hairpin of the *Plasmodium* ligands superimposes less well in the different AMA1-RON2 complexes (S2 Fig). This is most accentuated between residue Thr2055 in the tip of the PvRON2sp1 β-hairpin turn and the equivalent residue Arg2041 in PfRON2sp1, where the Cα positions differ by 1.9 Å in the respective homocomplexes. By contrast, Thr2055 (PvRON2sp1) in the heterocomplex is much closer to Arg2041 (PfRON2sp1) in the *P*. *falciparum* homocomplex as the Cα positions differ by only 0.6 Å, clearly illustrating that the *P*. *vivax* ligand partially adapts to the PfAMA1 binding site. These variations can be attributed largely to structural differences between the region Ser168-Phe169-Val170 in PvAMA1 and the equivalent region Asn223-Met224-Asn225 in PfAMA1, both of which contact Thr2055 of PvRON2sp1 in the homo- and hetero-complexes of this ligand.

### Species cross-reactivity of RON2 with AMA1

**Fig 5:**
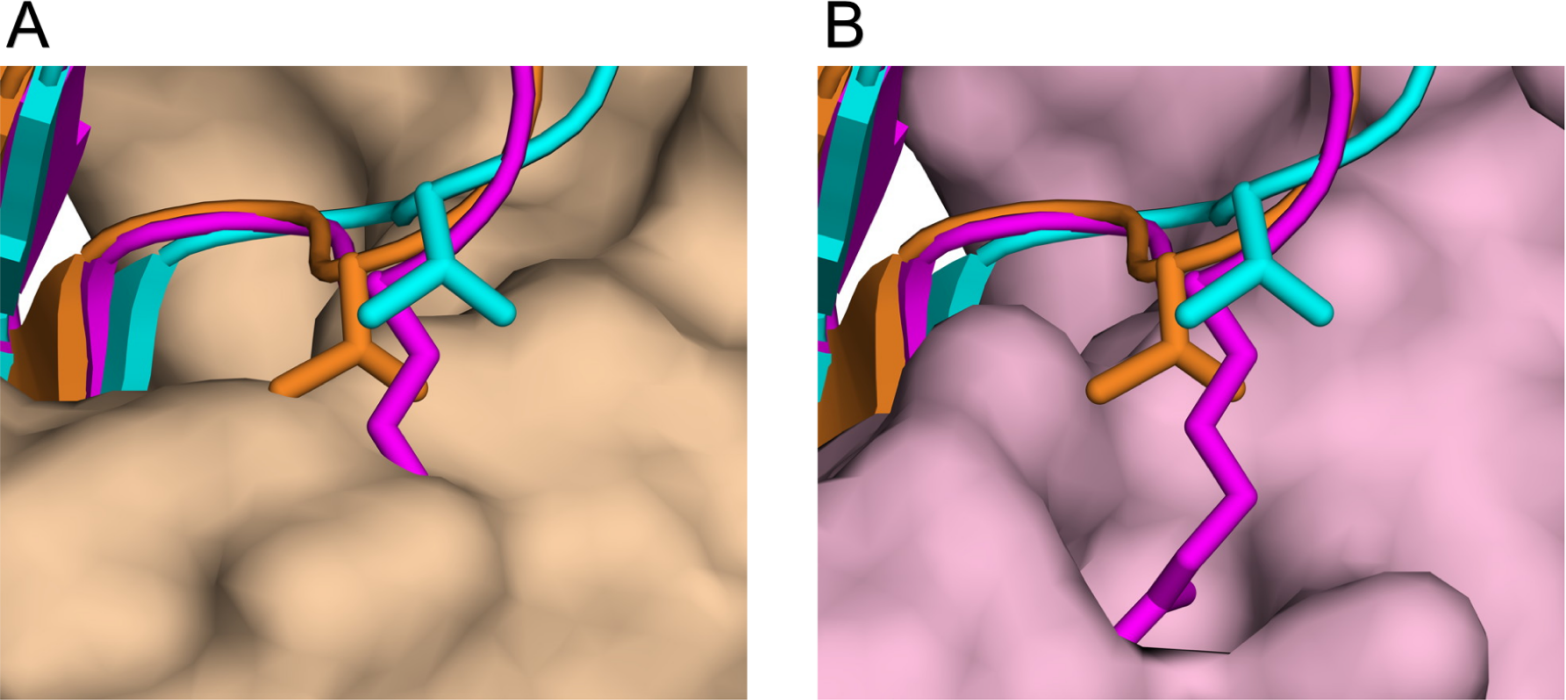
The critical role of Arg2041 in specific binding to PfAMA1. View of the disulphide-linked β-hairpin of the peptides in ribbon representation after superposition of PfAMA1-PvRON2sp1 and PfAMA1-PfRON2sp1 onto PvAMA1-PvRON2sp1 using the AMA1 coordinates only. PvRON2sp1 is shown in cyan for the homo-complex and in orange for the hetero-complex; PfRON2sp1 from the PfAMA1-PfRON2sp1 homo-complex is in magenta. The side chains of Arg2041 of PfRON2sp1 and the equivalent residue in PvRON2sp1, Thr2055, are also shown. (A) View of the superimposed peptides and PfAMA1 from the homo-complex in surface representation (pink). (B) The equivalent view with PvAMA1 from the hetero-complex in surface representation (light brown).

Our SPR studies show that PvRON2sp1 significantly cross-reacts with PfAMA1 (Table 3). The converse, however, is not true as no detectable binding was observed between PfRON2 and PvAMA1 (S1 Fig), consistent with previous measurements by ELISA [8]. The crystal structures of the hetero- and homocomplexes suggest that the lack of cross-reactivity between PvAMA1 and PfRON2 is largely due to residue Arg2041 in the latter component. In the *P*. *falciparum* complex, Arg2041 is buried in a pocket of the binding site (Fig 5A), forming seven hydrogen bonds, five from the buried guanidyl group and two from the main-chain amide. Indeed, this pocket is also occupied by arginine in the complexes formed between PfAMA1 and the antibody IgNAR [29] or the peptide R1 [10], both of which are invasion-inhibitory. In both cases, the arginine side chain superimposes very closely with Arg2041 [10], underlining the specificity of the pocket for arginine. Although this pocket is absent in the PvAMA1 crystal structure, the smaller side chain of the equivalent PvRON2 residue, Thr2055, is accommodated within the flatter topology of this region of the binding site but is positioned further out relative to Arg2041 in the PfAMA1-PfRON2sp1 homocomplex and, indeed, to Thr2055 in the *P*. *falciparum* heterocomplex (Fig 5B).

This comparison, however, requires further analysis as intermolecular contacts in the crystal lattice of the PvAMA1-PvRON2sp1 and apo PvAMA1 structures influence the conformation in this region. The arginine-specific pocket that accommodates PfRON2sp1-Arg2041 is bounded on one side by the side chain of PfAMA1-Lys235, which projects outwards into the solvent region. By contrast, the side chain of PvAMA1-Arg180, the residue equivalent to PfAMA1-Lys235, is constrained closer to the protein surface by contacts with a symmetry-related molecule, closing off the pocket. This is also the case for apo PvAMA1 since the two structures have similar molecular packing arrangements in the crystal lattice. In the absence of this packing constraint, modeling shows that the Arg180 side chain can indeed adopt a conformation similar to that of Lys235 in PfAMA1 to expose the binding pocket. Nonetheless, superposition of the two homo-complexes reveals that the Cα positions of PvRON2sp1-Thr2055 and PfRON2sp1-Arg2041 differ by 1.9 Å because of differences in surface topology in the hydrophobic groove of the two AMA1 homologues (see above). Consequently, the substitution by arginine at PvRON2sp1-Thr2055, although not sterically hindered, would not result in the optimal insertion of the side chain to form the five specific H-bonds, as in the PfAMA1-PfRON2sp1 homocomplex. Furthermore, the charged guanidyl group of Arg2041 would be in an unfavorable hydrophobic environment as it would pack against Leu170 of PvAMA1 (S3 Fig). These structural constraints on the PfRON2 ligand in the PvAMA1 hydrophobic groove could thus largely account for the lack of cross-reactivity between PfRON2 and PvAMA1.

## Conclusion

The formation of a MJ at the interface between outer membranes of the invading parasite and the host cell, in which AMA1 and RON2 play a key role, is probably a common feature across the *Apicomplexa* phylum as these two parasite protein components have been found in *Plasmodium*, *Toxoplasma*, *Babesia* and *Neospora*. Sequence and structural data from different *Plasmodium* AMA1 and RON2 proteins confirm that their interaction is highly conserved across this genus of the *Apicomplexa* phylum and that these two components have co-evolved within the different species. Our present results show that between *P*. *falciparum* and *P*. *vivax*, certain intermolecular contacts, both polar and nonpolar, are common to the AMA1/RON2 pair while others are particular to a given species, as the RON2 ligand adapts to sequence differences in the AMA1 binding groove. PvRON2 is able to bind to both PvAMA1 and PfAMA1 in spite of some differences in the intermolecular interactions. PfRON2, by contrast, does not bind to PvAMA1, which we attribute essentially to the unfavourable environment of Arg2041 in the PvAMA1 binding site. The higher affinity of PfRON2 for PfAMA1 (~10–20 nM) is probably due to the extensive polar and apolar interactions of Arg2041 in the binding pocket. ELISA-based binding studies of PfAMA1 to a GST fusion protein carrying the PfRON2sp1 peptide showed no detectable binding when Arg2041 was mutated to alanine [10]. Of note, the equivalent residue in the RON2 homologue of the closely related *P*. *reichenowi* is also arginine; in other species the equivalent residue is threonine or, in the case of the rodent homologues, leucine (S4 Fig). Thus, while co-evolution of plasmodial AMA1 and RON2 has occurred within species, our results show that small changes in sequence lead to loss of cross-reactivity in the case of PfRON2.

## Supporting information

S1 Fig Comparison of PfRON2sp1 binding to PfAMA1 and PvAMA1 by surface plasmon resonance. Binding was measured using PfRON2 at 1 μM concentration. SPR signal was obtained after subtraction of the control flow cell from the ligand flow cell and buffer injection from the analyte response.
(TIF)

S2 Fig Comparison of the PvRON2sp1 peptides in the homo- and hetero-complexes, and PfRON2sp1 in the PfAMA1-PfRON2sp1 homocomplex. (A) View of the peptides after superposition of PfAMA1-PvRON2sp1 (orange) and PfAMA1-PfRON2sp1 (magenta) (PDB entry 3ZWZ) onto PvAMA1-PvRON2sp1 (cyan) using the AMA1 coordinates only. The TgAMA1-TgRON2sp (PDB entry 2Y8T) is also included for comparison (green). The peptides are shown in ribbon representation with the side chains of the equivalent residues Thr2055 (PvRON2sp1), Arg2041 (PfRON2) and Ile1318 (TgRON2) shown in stick representation as in Fig 3. (B) View of the superimposed ligands rotated by 90° about a horizontal axis.
(TIF)

S3 Fig Binding pocket on PfAMA1 for Arg2041 of PfRON2. PfAMA1 is shown in surface representation with carbon atoms in green, oxygen in red and nitrogen in blue. The PfRON2sp1 peptide is in mauve with the Arg2041 side chain indicated. The position of the PvRON2sp1 peptide, coloured cyan, is shown after superposition of the PfAMA1 and PvAMA1 moieties of the *P*. *falciparum* and *P*. *vivax* homocomplexes. Thr2055 of PvRON2sp1 (residue equivalent to Arg2041 of PfRON2sp1) has been mutated to arginine and modelled to penetrate into the binding pocket of Arg2041. The guanidyl group of the modelled mutation Thr2055->Arg is unable to optimally penetrate into the pocket to form the 5 hydrogen bond interactions of Arg2041 observed in the *P*. *falciparum* homocomplex and, furthermore, is positioned in an apolar environment. This model is consistent with the lack of binding between PfRON2sp1 and PvAMA1.
(TIF)

S4 Fig Comparison of the RON2sp1 sequences from different *Plasmodium species* and *T. gondii*. PvRON2, *P*. *vivax*; PkRON2, *P*. *knowlesi*; PfRON2, *P*. *falciparum*, Pr, *P*. *reichenowi*; PoRON2, *P*. *ovale*; PgRON2, *P*. *gallinacium*; PbRON2, *P*. *berghei*; PyRON2, *P*. *yeoli*; PcRON2, *P*. *chabaudi*; TgRON2, *T*. *gondii*. The arrow indicated the alignment for residues equivalent to Thr2055 of PvRON2. Invariant residues are highlighted in cyan and highly conserved residues are highlighted in yellow. The column aligning residues with the critical residue Arg2041 from PfRON2 is indicated by an arrow.
(TIF)

S1 Table Comparison of AMA1-RON2 H-bonds. (DOCX)
