# Supplementary figures and images for: Cross-reactivity between apical membrane antgen 1 and rhoptry neck protein 2 in P. vivax and P. falciparum: A structural and binding study
Source: PLoS One. 2017 Aug 17;12(8):e0183198. doi: 10.1371/journal.pone.0183198 (PMC5560645; doi:10.1371/journal.pone.0183198)

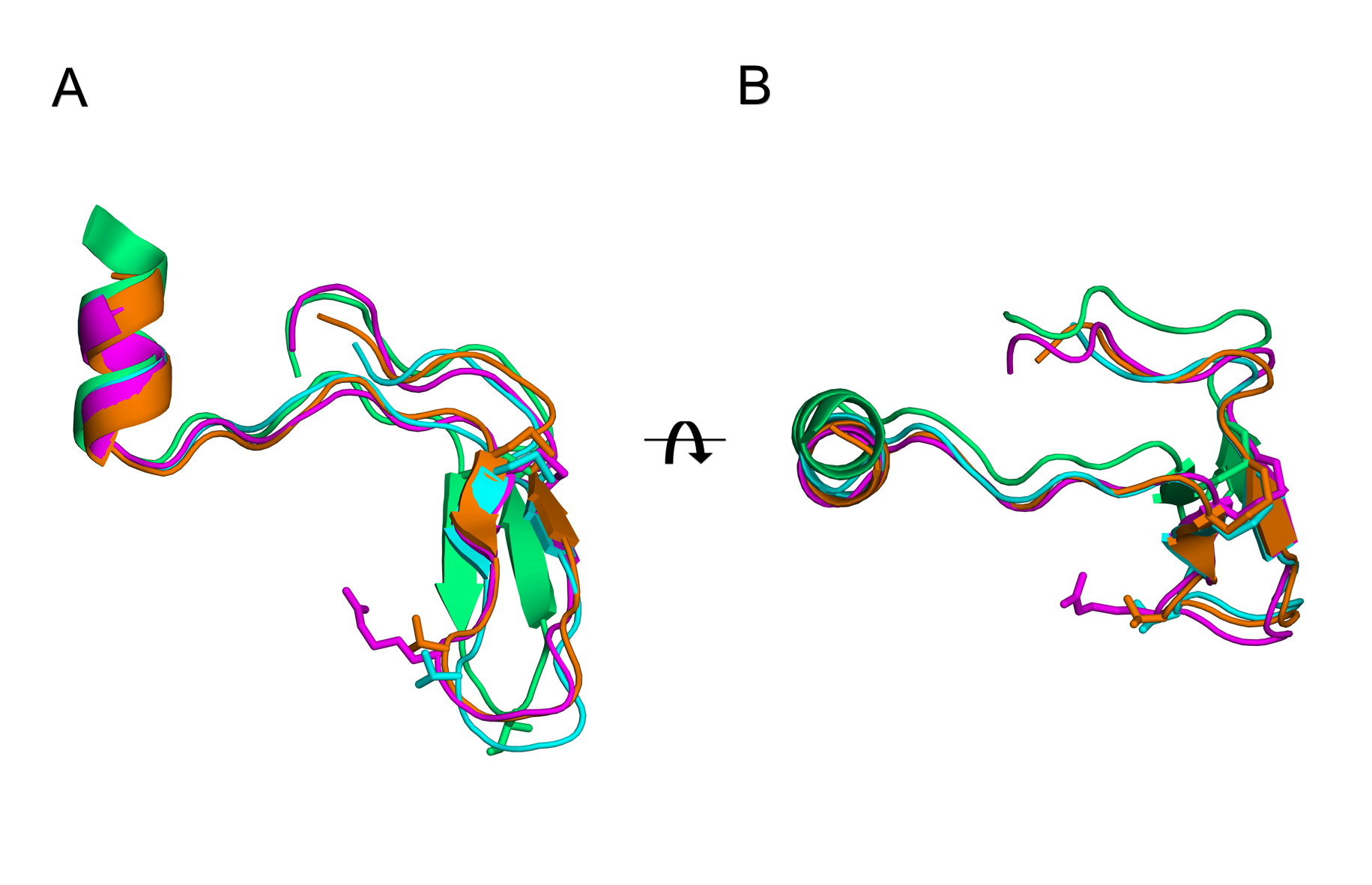

Supplement: S2 Fig — (A) View of the peptides after superposition of PfAMA1-PvRON2sp1 (orange) and PfAMA1-PfRON2sp1 (magenta) (PDB entry 3ZWZ) onto PvAMA1-PvRON2sp1 (cyan) using the AMA1 coordinates only. The TgAMA1-TgRON2sp (PDB entry 2Y8T) is also included for comparison (green). The peptides are shown in ribbon representation with the side chains of the equivalent residues Thr2055 (PvRON2sp1), Arg2041 (PfRON2) and Ile1318 (TgRON2) shown in stick representation as in Fig 3. (B) View of the superimposed ligands rotated by 90° about a horizontal axis. (TIF) [file pone.0183198.s002.tif]

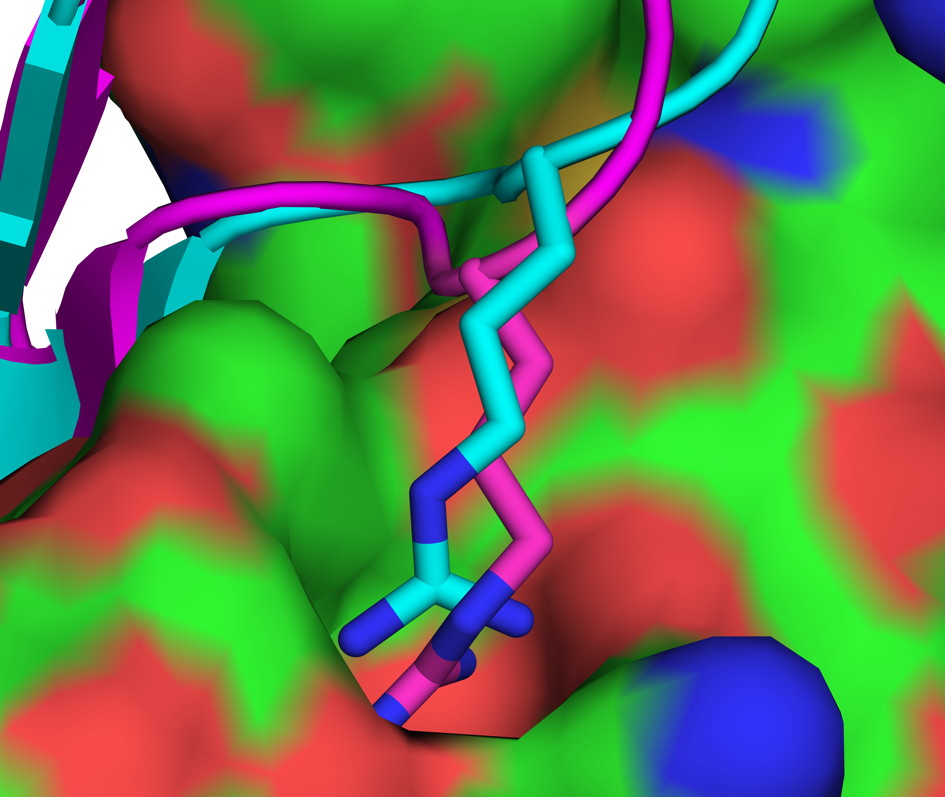

Supplement: S3 Fig — PfAMA1 is shown in surface representation with carbon atoms in green, oxygen in red and nitrogen in blue. The PfRON2sp1 peptide is in mauve with the Arg2041 side chain indicated. The position of the PvRON2sp1 peptide, coloured cyan, is shown after superposition of the PfAMA1 and PvAMA1 moieties of the P. falciparum and P. vivax homocomplexes. Thr2055 of PvRON2sp1 (residue equivalent to Arg2041 of PfRON2sp1) has been mutated to arginine and modelled to penetrate into the binding pocket of Arg2041. The guanidyl group of the modelled mutation Thr2055->Arg is unable to optimally penetrate into the pocket to form the 5 hydrogen bond interactions of Arg2041 observed in the P. falciparum homocomplex and, furthermore, is positioned in an apolar environment. This model is consistent with the lack of binding between PfRON2sp1 and PvAMA1. (TIF) [file pone.0183198.s003.tif]

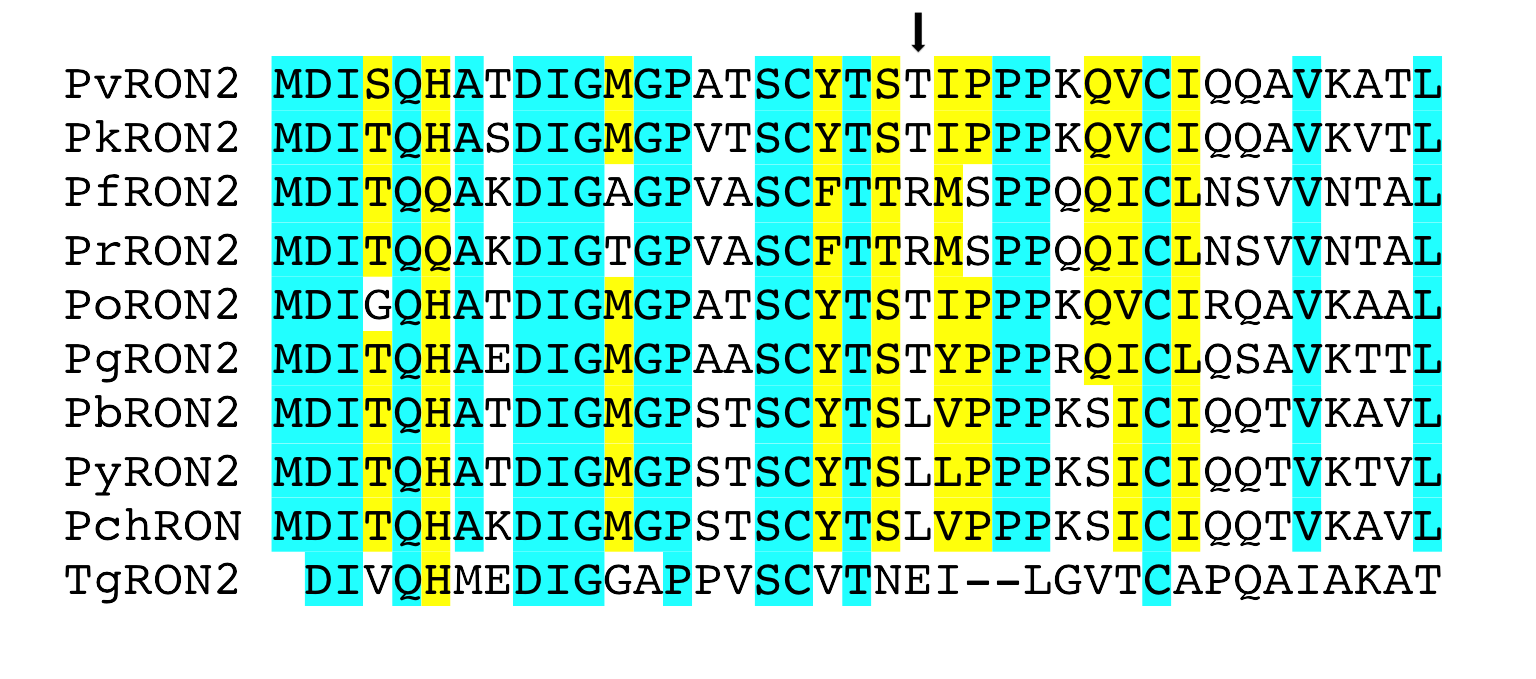

Supplement: S4 Fig — PvRON2, P. vivax; PkRON2, P. knowlesi; PfRON2, P. falciparum, Pr, P. reichenowi; PoRON2, P. ovale; PgRON2, P. gallinacium; PbRON2, P. berghei; PyRON2, P. yeoli; PcRON2, P. chabaudi; TgRON2, T. gondii. The arrow indicated the alignment for residues equivalent to Thr2055 of PvRON2. Invariant residues are highlighted in cyan and highly conserved residues are highlighted in yellow. The column aligning residues with the critical residue Arg2041 from PfRON2 is indicated by an arrow. (TIF) [file pone.0183198.s004.tif]
