# Supplementary material for: Cross-reactivity between apical membrane antgen 1 and rhoptry neck protein 2 in P. vivax and P. falciparum: A structural and binding study
Source: PLoS One. 2017 Aug 17;12(8):e0183198. doi: 10.1371/journal.pone.0183198 (PMC5560645; doi:10.1371/journal.pone.0183198)
Supplement: S1 Table — (DOCX) [file pone.0183198.s005.docx]

**Table S1. Comparison of AMA1-RON2 H-bonds**

| **PvRON2sp1-PvAMA1** | **PvRON2sp1-PfAMA1(FVO)** | **PfRON2sp1-PfAMA1(3D7)** |
| --- | --- | --- |
| 2039(HIS) NE2 202(ASN) OD1 3.01 | 2039(HIS) NE2 257(ASN) ND2 3.01 |  |
| 2042(ASP) O 88(ARG) NH1 2.92 | 2042(ASP) O 143(ARG) NH1 2.82 | 2028(ASP) O 143(ARG) NH1 2.83 |
| 2049(THR) N 196(TYR) OH 3.43  2049(THR) OG1 196(TYR) OH 2.83 | 2049(THR) N 251(TYR) OH 3.46  2049(THR) OG1 236(TYR) OH 3.26 | 2035(ALA) N 251(TYR) OH 3.42 |
|  |  | 2036(SER) N 227(ASP) OD2 3.46 |
| 2051(CYS) O 172(ALA) N 2.74 | 2051(CYS) O 227(ASP) N 2.94 | 2037(CYS) O 227(ASP) N 3.06  227(ASP) OD2 3.38  2037(CYS) N 227(ASP) OD2 2.80 |
| 2052(TYR) OH 181(HIS) NE2 2.75 | 2052(TYR) OH 236(TYR) OH 3.01 |  |
| 2053(THR) N 170(VAL) O 2.78  2053(THR) O 170(VAL) N 3.00 | 2053(THR) N 225(ASN) O 2.95  227(ASP) OD1 3.26  2053(THR) O 225(ASN) N 2.93  2053(THR) OG1 227(ASP) OD1 2.70 | 2039(THR) N 225(ILE) O 3.10  2039(THR) O 225(ILE) N 2.69 |
| 2054(SER) OG 168(SER) OG 3.14 |  |  |
| 2055(THR) N 168(SER) O 3.15  2055(THR) OG1 168(SER) O 2.94 | 2055(THR) N 223(ASN) O 3.02  223(ASN) OD1 2.95  2055(THR) OG1 222(GLY) O 3.44  223(ASN) O 2.79 | 2041(ARG) N 223(ASN) O 2.81  223(ASN) OD1 2.96  2041(ARG) NH1 222(GLY) O 2.87  224(MET) O 3.34  234(TYR) O 3.33  2041(ARG) NH2 234(TYR) O 3.08  232(SER) O 2.70 |
|  | 2056(ILE) N 223(ASN) OD1 2.91 | 2042(MET) N 223(ASN) OD1 2.84 |
| 2061(GLN) O 132(ASN) ND2 2.89  2061(GLN) O 132(ASN) ND2 3.06 |  |  |
| 2063(CYS) N 132(ASN) OD1 3.24  2063(CYS) O 132(ASN) N 2.96 |  | 2049(CYS) N 187(GLU) O 3.25  2049(CYS) O 187(GLU) N 2.97 |
| 2065(GLN) N 130(ASN) O 3.22 |  | 2051(ASN) N 185(PRO) O 3.19 |
| 2067(ALA) O 117(GLY) N 2.80 | 2067(ALA) O 172(GLY) N 2.78 | 2053(VAL) O 172(GLY) N 2.83 |
| 2069(LYS) N 115(ALA) O 3.00 | 2069(LYS) N 170(ALA) O 3.01 | 2055(ASN) N 170(ALA) O 2.80  2055(ASN) ND2 271(ASN) OD1 2.94  174(GLN) O 3.27 |
